# Asiatic Acid Inhibits OVX-Induced Osteoporosis and Osteoclastogenesis *Via* Regulating RANKL-Mediated NF-κb and Nfatc1 Signaling Pathways

**DOI:** 10.3389/fphar.2020.00331

**Published:** 2020-03-27

**Authors:** Guoju Hong, Lin Zhou, Xiaorui Han, Ping Sun, Zhenqiu Chen, Wei He, Jennifer Tickner, Leilei Chen, Xuguang Shi, Jiake Xu

**Affiliations:** ^1^The National Key Discipline and the Orthopedic Laboratory, Guangzhou University of Chinese Medicine, Guangzhou, China; ^2^School of Biomedical Sciences, the University of Western Australia, Perth, WA, Australia; ^3^Department of Endocrinology, the Fifth Affiliated Hospital of Guangzhou Medical University, Guangzhou, China; ^4^School of Medicine, South China University of Technology, Guangzhou, China; ^5^Department of Orthopedic, the First Affiliated Hospital of Guangdong Pharmaceutical University, Guangzhou, China; ^6^Department of Orthopedic, the First Affiliated Hospital of Guangzhou University of Chinese Medicine, Guangzhou, China; ^7^College of Chinese Materia Medical, Guangzhou University of Chinese Medicine, Guangzhou, China

**Keywords:** asiatic acid, osteoclast, RANKL, bone resorption, osteoporosis

## Abstract

*Asiatic acid* is a triterpenoid compound extracted from a medicinal plant *Centella asiatica*. It has been used as a highly efficient compound for the treatment of cancer and hyperlipidemia, as well as possessing potential antiinflammatory properties. However, its effects on bone metabolism and osteoporosis haven’t been reported. The purpose of our research were to reveal the biomolecular effects of *asiatic acid* on osteoclasts, and its underlying molecular mechanisms regulating its effects on receptor activator of NF-κB ligand (RANKL)-induced signaling pathways. We found that *asiatic acid* inhibited multinucleated tartrate-resistant acid phosphatase (TRAcP)-positive osteoclast differentiation and osteoclast induced bone loss. Real time PCR showed that *asiatic acid* reduced the expression of down-cascade target genes including *Ctsk*, *Nfatc1*, *Calcr*, and *Atp6v0d2*. Western blot and luciferase reporter gene assays revealed that *asiatic acid* inhibits RANKL mediated NF-κB and NFATc1 signalings. Further, *in vivo* study demonstrated *asiatic acid* attenuates estrogen deficiency-induced bone loss in ovariectomized mice. MicroCT and histology analyses revealed that osteoclast numbers were significantly suppressed in *asiatic acid* treated groups. Furthermore, serum levels of TRAcP and CTX-1 were downregulated in treated groups. Taken together, our data show that *asiatic acid* can inhibit osteoclastic formation and reduce OVX-induced bone resorption through RANKL-activated NF-κB or NFATc1 signaling, suggesting that *asiatic acid* may be a potential and effective natural compound for the therapy of excessive RANKL-related osteolytic diseases.

## Introduction

Osteolytic disease is a pathologic condition characterized by the imbalance of two coordinating and opposite aspects, bone formation by osteoblastogenesis and bone resorption by osteoclastogenesis (Goltzman, 2001; Kular et al., 2012). Maintaining the balance of such coupling is crucial to prevent the incidence of osteolytic disease (Manolagas and Jilka, 1995). When osteoclast induced bone loss markedly exceeds the normal level, then osteolytic disease occurs, which is manifested by bone mineral reduction and architectural deterioration in the skeletal system (Boyle et al., 2003; Caetano-Lopes et al., 2007).

The differentiation and formation of osteoclasts were regulated by several signaling pathways. One critical pathway is induced by the receptor activator of NF-κB ligand (RANKL) secreted mainly by osteocytes (Yasuda et al., 1998; Boyle et al., 2003). RANKL is necessary for osteoclast formation and bone mass resorption. Osteoclasts are multinucleated TRAcP-positive cells that are derived from bone marrow macrophages (BMM). RANKL binds with its receptor RANK and induces three key intracellular downstream signaling pathways; nuclear factor-κB (NF-κB), mitogen activated protein kinase (MAPK), and nuclear factor of activated T-cells (NFAT) (Baud’huin et al., 2007). Hence, targeting RANKL-activated downstream signaling pathways to alter osteoclast number and function is a promising way for multiple natural compounds to attenuate enhanced bone resorption and bone loss as previously reported (Tanaka et al., 2005).

*Asiatic acid*, a natural triterpenoid compound isolated from *Centella asiatica (L.) Urb*., has been identified as a potential therapeutic agent as it demonstrates antihyperlipidemia and antiinflammatory activities and also has shown efficacy against several types of cancer cells. It is reported that *asiatic acid* greatly inhibits endothelial hyperpermeability and suppresses the increased phosphorylation of IκB-α induced by TNF-α, contributing to the protection of human aortic endothelial cells from atherogenic stimuli (Fong et al., 2016). *Asiatic acid* also reduces the proliferation of human ovarian cancer cells by blocking the activation of the PI3K/Akt/mTOR pathway, as well as the proliferation of HepG2 cells by inhibiting the expression NDR1/2 kinase and enhancing the stability of p21WAF1/CIP1 protein (Chen et al., 2014; Ren et al., 2016). Furthermore, recent studies demonstrate that *asiatic acid* can attenuate neuroinflammation induced by various toxic agents *via* regulating Sirt1/NF-κB or NF-kB/STAT3/ERK signaling pathways (Park et al., 2017; Qian et al., 2018). *Asiatic acid* is also found to be effective in the treatment of cardiac hypertrophy through IL-1β-activated NF-κB signalling (Xu et al., 2015). Here, we hypothesize that *asiatic acid*, being related with the inhibition of NF-κB signaling, might have an undiscovered potential therapeutic effect on osteoporosis. Hence, we tried to evaluate the biofunction of *asiatic acid* on osteoclastogenesis and bone resorption *in vitro*. Furthermore, using oestrogen deficiency ovariectomized (OVX) animal model, we found that *asiatic acid* significantly reduces OVX induced bone loss, suggesting that *asiatic acid* is a candidate compound for the treatment of osteoporosis.

## Materials and Methods

### Materials and Reagents

*Asiatic acid* (HPLC > 98%) was purchased from the TransMIT (Gießen, Germany) and the compound at powder status was thawed in stock solution with dimethyl sulfoxide (DMSO, Sigma-Aldrich, St. Louis, MO, USA). Alpha–Minimal Essential Medium (α-MEM) and fetal bovine serum (FBS) as well as Trizol reagent were ordered from Life Technologies (Sydney, Australia). Anti-phosphate-ERK1/2, ERK1/2, anti-IkB α, anti-NFATc1, anti-c-Fos, and anti-Cathepsin K were purchased from Santa Cruz Biotechnology (Dallas, CA, USA). The loading control, anti-β-actin was obtained from Cell Signaling Technology (Danvers, MA, USA). The expression and purification of Glutathione S-transferase (GST)-rRANKL (GST-rRANKL) recombinant protein were performed as described (Xu et al., 2000). Macrophage colony stimulating factor (M-CSF) and ELISA kits of *TRAcP* and *CTX-1* were obtained from R & D company (Minnneapolis, MN, USA).

### *In Vitro* Osteoclastogenesis Assay

For *in vitro* osteoclastogenesis assay, BMMs were isolated from the long bones of C57BL/6J mice (Female, 12 weeks, weight 20 g, sourced from the Jackson Laboratory). The mice euthanized protocol was conducted with the approval obtained from the Animal Ethics Committee of the University of Western Australia (No. RA/3/100/1244). BMMs were incubated as 6×10^3^ cells per well in complete α-MEM (supplemented with 10% FBS and penicillin-streptomycin) as well as M-CSF (25 ng/ml), and cultured in 5% CO_2_ at 37 °C overnight until confluent. The next day, BMMs were starved for 4 h and further stimulated with GST-rRANKL (50 ng/ml) with or without various concentrations of *asiatic acid* (0.5, 1, 2.5, 5, 10, 20 µM) for every two days until multinucleated osteoclasts observed. BMMs were fixed with 4% paraformaldehyde in phosphate buffered saline (PBS) for 10 min and stained for enzymatic activity of tartrate resistant acid phosphatase (TRAcP) using the leukocyte acid phosphatase staining kit (Sigma, St. Louis, MO, USA). TRAcP-positive cells (nucleus ≥3) were recorded as osteoclast-like cells.

### Cytotoxicity Assay for Cells Proliferation and Viability

For the detection of cytotoxicity of *asiatic acid*, BMMs were cultured at 6×10^3^ cells per well in complete α-MEM with M-CSF for 12 h incubation (condition as 5% CO2 at 37°C). Different concentrations of *asiatic acid* were then added to BMMs with the complete α-MEM with M-CSF for total two days. After that, 20 µl MTS solution (Promega, Sydney, Australia) was added to each well for 2 h. The optical density (OD) manifested by the MTS solution was measured by a BMG plate reader (Thermo Labsystem Multiscan Spectrum, Thermo Fisher, Waltham, MA, USA) with 490 nm wavelength.

### Bone Resorption Assay in Hydroxyapatite Plate

BMMs were cultured on to 6-well plates at a concentration of 1×10^5^ cells per well overnight, and then stimulated with 50 ng/ml GST-rRANKL and corresponding M-CSF for 4-day incubation. Once mature osteoclasts emerged, the cells were smoothly detached from the plates by cell dissociation solution (Sigma, St. Louis, MO, USA). Cell number was then counted, and same numbers of osteoclast-like cells were plated into a hydroxyapatite-coated plate (Corning, Inc., Corning, NY, USA). After seeding onto the hydroxyapatite plate, the cells were intervened by *asiatic acid* at various concentrations (10 and 20 µM) with GST-rRANKL and M-CSF. After 48 h, TRAcP staining was used to assess the number of osteoclast-like cells for half of the cells. The other half were imaged directedly and the hydroxyapatite resorption area was analyzed by ImageJ software. The final result was manifested by the percentage of resorbed area (ratio of osteoclasts number and hydroxyapatite resorption area).

### RNA Extraction and Real-Time Polymerase Chain Reaction

For real-time polymerase chain reaction (RT-PCR), freshly isolated BMMs from C57BL/6 mice were seeded at a concentration of 1×10^5^ cells per well. Cells were incubated in complete α-MEM with M-CSF and GST-rRANKL as well as *asiatic acid* at two concentrations (5 and 10 µM) for 5 days. Cells were lysed when osteoclasts were formed, and total RNAs was isolated and obtained from the lysed cells by Trizol reagent. Single-stranded cDNA was reverse transcribed from 1 µg total RNA. Specific primers used are shown in **Table 1**. qPCR reactions were then measured in a ViiA RT-PCR (Applied Biosystems, Warrington, UK). PCR amplifications were performed based on the following program: 94°C for 5 min, followed by 30 cycles of 94°C for 40 s, 60°C for 40 s, and 72°C for 40 s, and a final extension step of 5 min at 72°C. The comparative 2^–ΔΔCT^ values normalized to control samples were presented.

**Table 1:** Sequences of both the forward and reverse primers of mRNAs in RT-qPCR.

Genes	Forward (5’-3’)	Reverse (5’-3’)
*Ctsk*	GGGAGAAAAACCTGAAGC	ATTCTGGGGACTCAGAGC
*Calcr*	TGGTTGAGGTTGTGCCCA	CTCGTGGGTTTGCCTCATC
*Nfatc1*	CAACGCCCTGACCACCGATAG	GGCTG CCTTCCGTCTCATAGT
*Atp6v0d2*	ATGCTTGAGACTGCAGAG	TTATAAAATTGGAATGTAGCT
*Gapdh*	ACCACAGTCCATGCCATCAC	TCCACCACCCTGTTGCTGTA

Ctsk, cathepsin K; Calcr, Calcitonin receptor; Nfatc1, nuclear factor of activated T-cells cytoplasmic 1; Atp6v0d2, ATPase H+ Transporting V0 Subunit D2; Gapdh, Glyceraldehyde-3-Phosphate Dehydrogenase.

### Luciferase Report Gene Assay for NF-κB and NFAT

In order to investigate the transcriptional activity of NF- κB and NFAT, luciferase reporter gene assays were performed. Briefly, RAW264.7 cells were transfected with an NF- κB or an NFAT luciferase reporter construct. Then the transfected cells were cultured onto a 48-well plate at the density of 1.5× 0^5^ or 5× 10^4^ cells per well respectively (Wang et al., 2003; van der Kraan et al., 2013). Cells were pretreated with *asiatic acid* at 2.5, 5, 10, and 20 µM concentration for 1 h, and then incubated with 50 ng/ml GST-rRANKL. After 6 h for NF-κB reporter cells or 24 h for NFAT reporter cells, both cells were lysed for 20-min centrifugation at 14,000g at 4°C. Luciferase activity of NF-κB and NFAT was assessed by a Luciferase Assay machine (Promega, Sydney, Australia) and evaluated by a BMG Optima luminescence reader (BMG LABTECH GMBH, Ortenberg, Germany).

### Evaluation of Ca^2+^ Oscillation in Osteoclast

BMMs were cultured at a concentration of 1× 10^4^ cells per well in 48-well trays with complete α-MEM nutritional solution. Next day, 10 µM *asiatic acid* with 50 ng/ml GST-rRANKL and 25 ng/ml M-CSF was added and cells was incubated for one day. After washed by HANKS balanced salt solution (supplemented with 1 mM probenecid and 1% FBS), the cells were stained by 4 mM Fluo4 solution (Fluo4-AM dissolved in 20% pluronic-F127) (Molecular probes, Thermo Fisher Scientific, Scoresby, Australia) at 37°C for 45 min. Staining solution was removed and cells were maintained in HANKS solution at room temperature for 20 min. Cells were washed prior to fluorescence signals being detected by a fluorescent microscope at a wavelength of 488 nm. Continued images were recorded every 3 seconds for 1 min and Ca^2+^ flux quantified by a Nikon Basic Research Software. Calcium oscillation intensity was calculated and presented as the interval between the maximum and minimum of fluorescence value within the scopes.

### Western Blot Assay

BMMs were cultured at a density of 5×10^5^ cells per well with complete α-MEM medium. For testing the expression of p-ERK, ERK, IkB α, and β-actin, cells were added first by *asiatic acid* for 1 h and then followed with RANKL at the time point as 0, 5, 10, 20, 30 and 60 min or 0, 1, 3, and 5 days for the detection of NFATc1 (Nuclear Factor of Activated T Cells 1), c-Fos, and β-actin expression. Cells were lysed and centrifuged (14,000 g, 5 min) to clarify the lysate. The bicinchoninic acid assays (BCA assay) were undertaken to qualify the amount of protein levels. Protein samples were separated using SDS-polyacrylamide gel electrophoresis (SDS-PAGE) and transferred to poly membranes (GE Healthcare, Silverwater, Australia). The membrane was blocked in TBS-Tween (TBST) diluted 5% skim milk for 1 h at room temperature and then added with specific primary antibodies, including p-ERK, ERK, IkB α, NFATc1, c-Fos, and β-actin antibodies (1:1,000) at 4°C, with shaking, overnight. The membrane was washed by TBST and subsequently incubated in appropriate horseradish peroxidase-conjugated secondary antibodies (1:5,000). Antibody reactivity was then detected using the enhanced chemiluminescence (ECL) reagents (Amersham Pharmacia Biotech, Australia). Finally, the image was acquired using an Imagequant LAS 4000 (GE Healthcare, Australia).

### OVX Mouse Model

Eighteen C57BL/6J female mice [12 weeks, weight 20 g, source from Guangzhou University of Chinese Medicine, No. SCXK (YUE) 2013-0001] tested for specific pathogens and housed in separate ventilated cages (IVCs) for a 12 h light/dark cycle. Mice were divided into three groups randomly, including sham group, OVX model group, and the OVX+ *asiatic acid* group. Subsequently, the OVX group and the OVX+*asiatic acid* group underwent ovariectomy, while the sham group received a sham operation without ovary dissection. At the time of surgery, the mice were anesthetized with 10% chloral hydrate solution. All mice received a 1-week wound recovery time after surgery. Then the mice in the OVX with *asiatic acid* group were given an intraperitoneal injection of *asiatic acid* at 3 mg/kg every 2 days. PBS containing 1% DMSO were injected intraperitoneally into the mice in the sham and OVX groups instead of *asiatic acid* as normal and negative controls. The mice were euthanized six weeks after the operation. For each mouse, their left femur was dissected for Micro-CT scanning and its right femur for histological examination.

### Micro-CT, Bone Histomorphometric Analyses, and Elisa Testing

The left femurs without muscle soft tissue were extracted from the treatment and control groups and fixed in 4% paraformaldehyde solution. The bone was maintained in a tube for Micro-CT scanning. Femur was scanned in a Bruker MicroCT Skyscan 1272 system (Kontich, Belgium) at 9 μm resolution. Each femur was imaged using the following settings and parameters: 70kV, 200µA, 10µm Al filter, 300 ms exposure, pixel size 8.89 mm, 0.4-degree rotation step. Three-dimensional (3D) Micro-CT images were then reconstructed depending on the constant threshold values. Measurement selected the proximal part of the femur under the growth plate as the region of interest (ROI). This tissue volume was adopted and transferred to the analyses program CTAn (Bruker microCT, Kontich, Belgium).

The right femurs were decalcified and embedded into paraffin blocks for sectioning and staining. Sequential 5-µm-thick slices were prepared and stained using hematoxylin and eosin, and also with immunohistochemical staining using an anti-Cathepsin K antibody. Full slices were scanned at a high resolution, and trabecular histological analyses were performed using BIOQUANT OSTEO software (Bioquant Image Analysis Corporation, Nashville, TN, USA).

In addition, blood was drawn from the abdominal aorta of mice in each group and centrifuged to obtain serum of mice. Serum levels of TRAcP and C-terminal telopeptide (CTX-1) were assessed using enzyme-linked immunosorbent assay (ELISA) kit.

### Statistical Analysis

The original data were presented as the form as mean ± standard deviation (SD) in the final result. Each experiment was performed independently for at least three times and triplicate values obtained from these experiments were collected. A *p* < 0.05 considered statistically significant was determined by Student’s *t* test.

## Results

### Asiatic Acid Attenuates RANKL-Mediated Osteoclastogenesis

**Figure 1:**
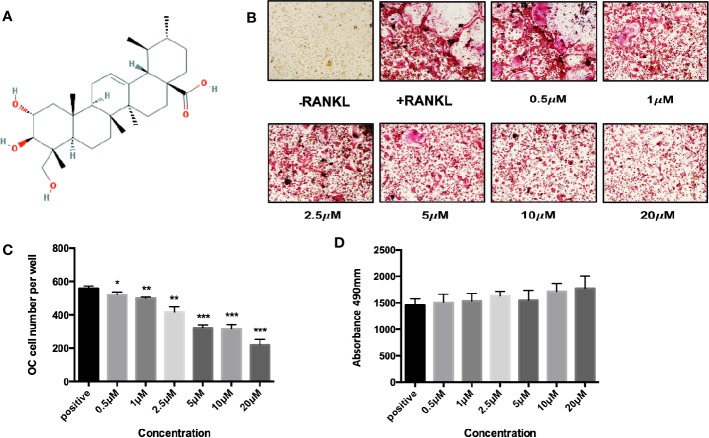
*Asiatic acid* inhibits receptor activator of NF-κB ligand (RANKL)–induced osteoclastogenesis without altering cell survival. **(A)** Chemical structure of *asiatic acid* with a triterpenoid feature (cited from PubChem substance CID 119034). **(B)** Representative images showing the dose-dependent inhibition of osteoclastogenesis following treatment with *asiatic acid*, with an IC_50_ at 10 μ μM. Scale bar = 10 μm; **(C)** Quantification of the number of tartrate-resistant acid phosphatase (TRAcP)–positive multinuclear cells (containing three or more nuclei) following treatment with *asiatic acid*. **(D)** BMMs were treated with different concentrations of *asiatic acid* for 48 h and cell survival was measured by MTS assay. (*p value <0.05, **p value <0.01, ***p value <0.001, versus RANKL-treated control).

To explore the biofunction of the natural compound *asiatic acid* (**Figure 1A**) on the differentiation of osteoclasts, osteoclastogenesis assays were utilized. The results indicated that treatment with *asiatic acid* led to a dose-dependent inhibition of RANKL-induced osteoclastogenesis. *asiatic acid* significantly reduced osteoclastogenesis at doses which were higher than 5 µM (**Figures 1B, C**). In order to examine the cell viability of *asiatic acid*–treated BMMs, an MTS cell survival assay was performed. *asiatic acid* showed no cytotoxic effects on BMMs at a dose of 20 μM or lower (**Figure 1D**).

### Asiatic Acid Inhibits Osteoclastic Bone Resorption

**Figure 2:**
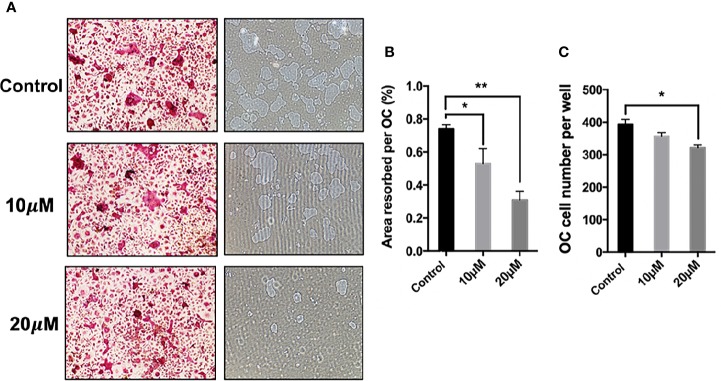
*Asiatic acid* suppresses receptor activator of NF-κB ligand (RANKL)–induced hydroxyapatite bone resorption. **(A)** Representative images demonstrating the areas of hydroxyapatite resorption and the number of tartrate-resistant acid phosphatase (TRAcP)–stained osteoclasts. Scale bar = 500 μm; **(B)** Analysis of TRAcP positive multinucleated cells (containing three or more nuclei) number after treatment with *asiatic acid*; **(C)** Percentage of the area of hydroxyapatite surface resorbed per osteoclast following treatment with *asiatic acid* (*p value <0.05, **p value <0.01, compared with control group).

To identify the potential effect of *asiatic acid* on bone resorption, hydroxyapatite-coated plates were used. Our findings suggested that, compared with the normal control group, the percentage of resorbed area per multinucleated osteoclast was attenuated if osteoclasts were exposed to *asiatic acid* at 10 and 20 μM. As shown in **Figure 2**, a slight reduction in osteoclast number was also noticed in *asiatic acid–*treated osteoclasts, however, such reduction is obviously lesser than the decrease of resorbing area. Our results indicated that *asiatic acid* suppresses osteoclast resorptive function.

### Asiatic Acid Suppresses RANKL-Induced Gene Expression

**Figure 3:**
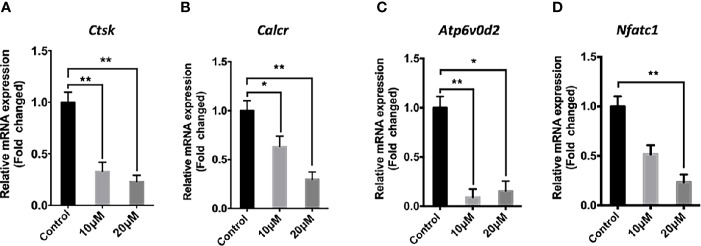
*Asiatic acid* inhibits receptor activator of NF-κB ligand (RANKL)–induced gene expression in bone marrow macrophages (BMMs). RT-qPCR was used to evaluate the expression of genes in the RANKL signaling pathway in BMM cells plated with complete Alpha–Minimal Essential Medium (α-MEM) with macrophage colony stimulating factor (M-CSF) and Glutathione S-transferase–rRANKL (GST-rRANKL) (50 ng/ml) in the presence or absence of *asiatic acid* at 10 and 20 μ μ M. Gene expression was normalized to Glyceraldehyde-3-Phosphate Dehydrogenase (*Gapdh*); **(A)** cathepsin K (*Ctsk*); **(B)** Calcitonin receptor (*Calcr*); **(C)** ATPase H+ Transporting V0 Subunit D2 (Atp6v0d2); **(D)** nuclear factor of activated T-cells cytoplasmic 1 (Nfatc1). (* *p* < 0.05, ** *p* < 0.01 relative to RANKL-stimulated controls).

The effect of *asiatic acid* on RANKL-induced downstream gene expression was examined during osteoclastogenesis by real time PCR. Consistent with osteoclast formation and bone resorption activity assays, the expression of osteoclast marker genes includi*ng* cathepsin K (*Ctsk*), Calcitonin receptor (*Calcr*), nuclear factor of activated T-cells cytoplasmic 1 (*Nfatc1*), and ATPase H+ Transporting V0 Subunit D2 (*Atp6v0d2*), decreased significantly following *asiatic acid* treatment, in a dose-dependent manner (**Figures 3A–D**).

### Asiatic Acid Inhibits RANKL-Induced Intracellular Ca^2+^ Oscillations

**Figure 4:**
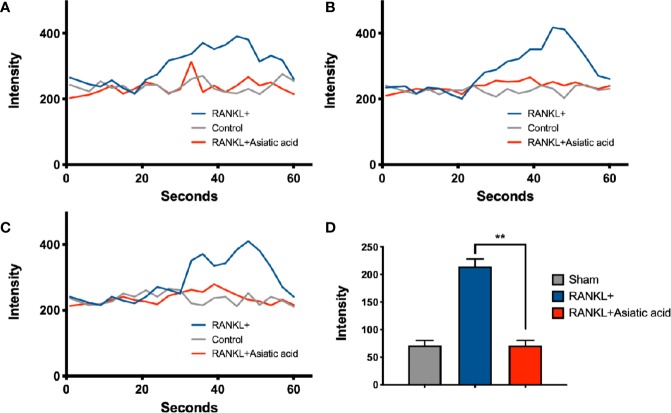
*Asiatic acid* abrogates receptor activator of NF-κB ligand (RANKL)–induced Ca^2+^ oscillation. Bone marrow macrophages (BMMs) were cultured with Glutathione S-transferase–rRANKL (GST-rRANKL) (50 ng/ml) and then calcium flux was tested using Fluo4 calcium indicator. **(A–C)** Representative graphs showing individual cell calcium fluctuations for the groups: RANKL only, group treated with macrophage colony stimulating factor (M-CSF) only (sham group) and group treated with RANKL and *asiatic acid* (20 μ μ M). **(D)** Average change in calcium intensity per cell in each group (**p < 0.01, relative to only RANKL-treated group).

To determine the impact of *asiatic acid* on RANKL-induced Ca^2+^ signaling, a Fluo4-based intracellular Ca^2+^ oscillation assay was used. RANKL stimulation was shown to induce Ca^2+^ oscillations. Treatment with *asiatic acid* significantly inhibited the RANKL mediated Ca^2+^ oscillations (**Figure 4**). These results revealed that *asiatic acid* suppresses RANKL-induced Ca^2+^ oscillation in osteoclasts.

### Asiatic Acid Inhibits RANKL-Induced NF-κB Activation and Phosphorylation of ERK

**Figure 5:**
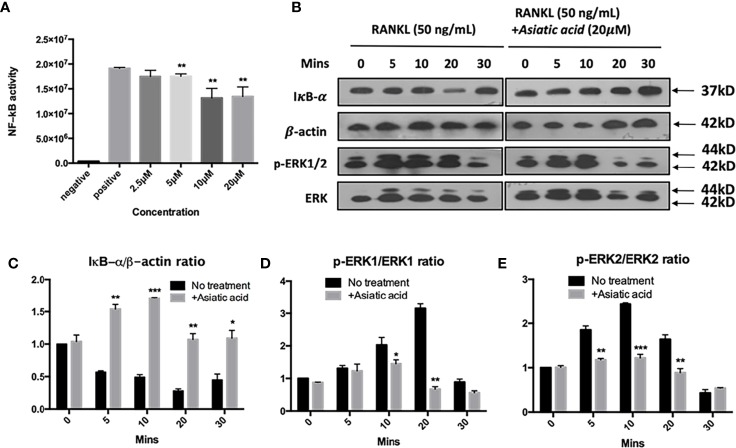
*Asiatic acid* abrogates receptor activator of NF-κB ligand (RANKL)–induced NF-κB activity, IκB-α degradation, and phosphorylation of extracellular a signal-regulated kinases (ERK). **(A)** Luciferase activity in RANKL stimulated NF-κB luciferase construct transfected RAW264.7 cells. Cells were pretreated with *asiatic acid* and then stimulated with GST-rRANKL (50ng/ml); **(B)** Protein lysates from bone marrow macrophages (BMMs)–induced osteoclasts pretreated with *asiatic acid* (20 20 µM), which were then stimulated with GST-rRANKL (50 ng/ml) for indicated time points. Western blot assay was performed using IκB-α, β-actin and p-ERK, ERK specific antibodies. Relative results were expressed as the ratio of the amount of protein relative to β-actin; IκB-α **(C)**, p-ERK1/ERK1 **(D)** or p-ERK2/ERK2 **(E)** as determined by ImageJ. (*p < 0.05, **p < 0.01, ***p < 0.001, relative to RANKL-treated, *asiatic acid*–untreated controls).

To determine the effects of *asiatic acid* on signaling pathways regulating osteoclast differentiation, NF-κB luciferase report gene transfected cells were pretreated with various doses of *asiatic acid* up to 20 μM and incubated for 1 h. After that, cells were incubated with GST-RANKL (50 ng/ml) for more 6 h. **Figure 5A** demonstrates that *asiatic acid* restrained NF-κB transcription activation from the concentration of 2.5μM to 20 μM in a dose-dependent manner. Additionally, BMMs were treated with 20 μM *asiatic acid* and incubated with RANKL for 0, 5, 10, 20, 30, and 60 min. Analysis by Western blot assay illustrated that *asiatic acid* at 10 μM markedly reduces I κB α degradation comparing to the untreated control group, but also suppresses RANKL-induced ERK phosphorylation relative to total ERK (**Figure 5B**). The maximum reduction of I κB α degradation was at 10 min (**Figure 5C**), while the maximum reduction of p-ERK1 (**Figure 5D**) and p-ERK2 (**Figure 5E**) was at 20 min. However, no significant inhibitory effect of asiatic acid was found on phosphorylation of P65 and P38 (**Figure S1**).

### Asiatic Acid Inhibits RANKL-Induced NFAT Activation

**Figure 6:**
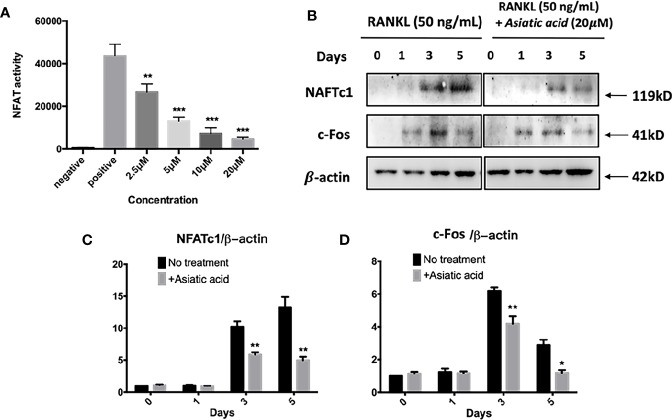
*Asiatic acid* abrogates receptor activator of NF-κB ligand (RANKL)–induced nuclear factor of activated T-cells cytoplasmic 1 (NFATc1) activity and protein expression. **(A)** Luciferase activity in RANKL stimulated NFAT luciferase construct transfected RAW264.7. Cells were treated with various concentrations of *asiatic acid*, and stimulated with GST-rRANKL (50 ng/ml) (*p < 0.05, **p < 0.01 relative to RANKL-stimulated controls); **(B)** Protein lysates from BMMs-induced osteoclasts pretreated with *asiatic acid* (20 μ μ M), which were stimulated with GST-rRANKL for the given times. Western blot assay was performed using NFATc1, c-Fos and β-actin specific antibodies. Relative results were expressed as the ratio of the amount of protein relative to β-actin; NFATc1 **(C)**, and c-Fos **(D)** determined by ImageJ. (*p < 0.05, **p < 0.01, relative to RANKL-treated, *asiatic acid*–untreated controls).

Next, luciferase reporter assay was performed to examine the effect of *asiatic acid* on NFAT transcription activation. The findings reveal that *asiatic acid* inhibited NFAT activity from the concentration of 2.5 μM to 20 μM in a dose-dependent manner (**Figure 6A**). Western blot assay was performed to investigate NFATc1 and c-Fos protein levels in BMMs stimulated with GST-RANKL (50 ng/ml) for 0, 1, 3, and 5 days with or without *asiatic acid*. *asiatic acid* greatly suppressed protein levels of NFATc1 and c-Fos (**Figure 6B**). The maximum reduction of NFATc1 protein expression was at day 3 (**Figure 6C**) whereas the maximum decease of c-Fos protein expression was at day 5 (**Figure 6D**).

### Asiatic Acid Inhibits Ovariectomy-Induced Bone Loss

**Figure 7:**
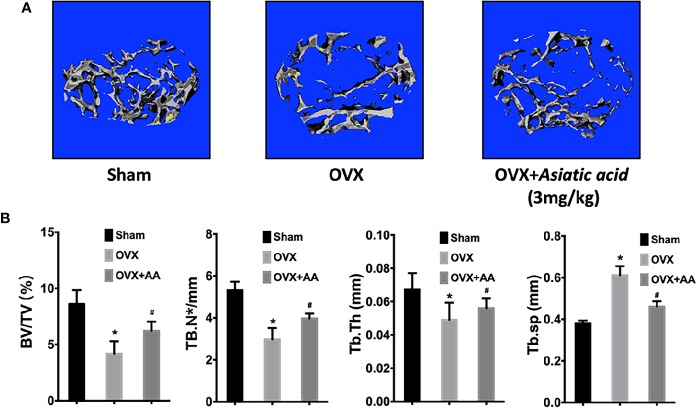
*Asiatic acid* inhibits oestrogen deficiency-induced bone resorption. **(A)** Representative three-dimensional (3D) reconstruction image and Micro-CT analysis of trabecular bone microarchitecture from the femur of sham mice, oestrogen deficiency (OVX) mice, and test group treated with *asiatic acid* at 3 mg/kg. The result shows the potential protective effect of *asiatic acid* in OVX-induced osteoporosis. **(B)** Quantitative analyses of bone volume/total volume (BV/TV), trabecular number (Tb.N), trabecular thickness (Tb.Th), and trabecular separation (Tb.Sp) (*p < 0.05 relative to controls group, ^#^*p* < 0.05 relative to OVX untreated group, AA is the abbreviation of *asiatic acid*).

No adverse events were observed during the OVX operation or the injection with *asiatic acid*. After treatment, the femurs were isolated and soft tissues were completely removed. The femurs from mice were then subjected to Micro-CT detection. Micro-CT analysis showed that *asiatic acid* treatment significantly reduced bone resorption associated with estrogen deficiency, as shown by the enhancement of BV/TV and reduction of Tb. Sp in *asiatic acid* treated mice when compared with the OVX mice (**Figure 7**). The data suggested that *asiatic acid* reduced OVX-induced bone loss.

**Figure 8:**
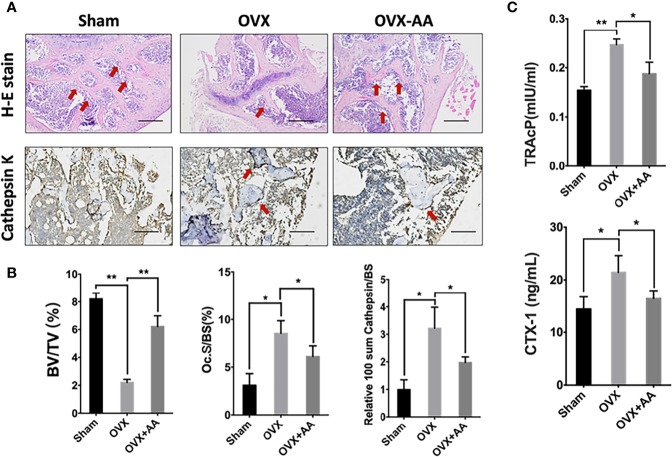
*Asiatic acid* prevents oestrogen deficiency (OVX) mouse model from bone loss *via* suppressing osteoclast activity. **(A)** Representative images of decalcified metaphysis stained with H&E and immunohistochemical staining of Cathepsin K on sham mice, OVX mice, and test group treated with 3 mg/kg *asiatic acid*. Red arrow indicates the trabecular and expression of *Cathepsin K* in the metaphysis. Mag 4X, scale bar: 500 mm. **(B)** Quantitative analyses of bone volume/total volume (BV/TV) and osteoclast number/bone surface (N.Oc/BS) as well as the relative Cathepsin K expression in mouse femur. **(C)** The serum levels of tartrate-resistant acid phosphatase (TRAcP) and C-terminal telopeptide (CTX-1) in each group (*p < 0.05 and **p < 0.01 relative to controls and OVX untreated controls, AA is the abbreviation of *asiatic acid*).

Bone histological study was further undertaken to analyze the impact of *asiatic acid* on OVX-induced bone loss. The results revealed a protective effect of BV/TV in the OVX+*asiatic acid* group relative to the OVX group (**Figures 8A, B**), being consistent with the Micro-CT results. Moreover, compared with the OVX group, osteoclast number (highlighted by Cathepsin K staining)/bone surface (N.Oc/BS) and the relative *Cathepsin K* expression were reduced in the *asiatic acid*–treated OVX mice. The serum levels of TRAcP and CTX-1 were reduced by *asiatic acid* as compared with the OVX model (**Figure 8C**). Our findings indicated that *asiatic acid* restrained estrogen deficiency bone loss by inhibiting osteoclast activity.

## Discussion

***Centella asiatica (L.) Urb*.** containing the main component *asiatic acid* has been prevalently used in treating acute or chronic inflammation of human organ systems in many ancient Asian countries, including China, Japan and Korea. Recently, *asiatic acid* has been found to display suppressive effects on cancer cells, inflammation, and cardiac hypertrophy (Chen et al., 2014; Xu et al., 2015; Park et al., 2017). Particularly, *asiatic acid* was able to modulate the NF-κB related signaling pathway in neuroinflammation (Park et al., 2017; Qian et al., 2018). In our study, the effects of *asiatic acid* on osteoclast formation and activity were explored, including its underlying molecular regulation in RANKL related signalling pathways, as well as its therapeutic effect on an OVX-induced estrogen deficiency bone loss mouse model.

Our results demonstrated that *asiatic acid* inhibited RANKL-induced osteoclastogenesis and osteoclast activity. According to the luciferase reporter result, *asiatic acid* was able to attenuate NF-κB transtription activity and suppress the level of osteoclast target genes consistent with its inhibitootry effect on osteoclast formation. During osteoclastogenesis, the RANKL-induced NF-κB and its downstream pathways are regarded as the main targets to be stimulated (Xu et al., 2009). The interaction between RANK and RANKL promotes the level of degradation of IκBα through the proteasome and NF-κB release (Mercurio and Manning, 1999; Brown et al., 2008). Through the activation of transcription of osteoclast-specific genes, NF-κB is critical to osteoclast formation and bone resorption (Soysa and Alles, 2009).

*Asiatic acid* also effectively attenuated NFAT activity and the level of NFATc1 protein. As previously reported, NFATc1 and its related factors are a crucial signal leading to osteoclast differentiation (Hirotani et al., 2004; Matsuo et al., 2004). RANKL-mediated Ca2^+^ oscillation leads to the activation of calcineurin, and auto amplification of NFATc1 (Kular et al., 2012). The reduction of Ca2^+^ signaling in osteoclasts and the expression level of NFATc1 protein following treatment with *asiatic acid* are important for osteoclast inhibition (Kajiya, 2012). NFAT signaling is regulated by the activity of NF- κB, hence the identified inhibition effect of NFATc1 by *asiatic acid* may be partially due to the attenuation of NF-κB activity (Takatsuna et al., 2005). The data demonstrated that target genes of NFATc1 activation, such as *Atp6v0d2* which was fundamental for osteoclast fusion, was inhibited, indicating that the reduced expression of NFATc1 was critical in the mechanism by which *asiatic acid* inhibited RANKL-induced osteoclastogenesis. *Asiatic acid* was also found to suppress ERK phosphorylation in the MAPK signaling pathway involved in osteoclast formation and survival, which was downstream from tumor necrosis factor receptor-associated factor 6 (TRAF6) (Miyazaki et al., 2000; Nakamura et al., 2003). Thus, the potential impact of *Asiatic acid* on survival of osteoclasts is multifactorial though the regulation of MAPK/ERK signaling pathways.

Consistent with the experimental results *in vitro*, the *in vivo* effect of *asiatic acid* in OVX mice showed that it can protect against bone loss through blocking osteoclastognesis. These preclinical experimental findings imply that *asiatic acid* might be an efficient drug prototype choice, and serve as a novel potential natural compound in suppressing osteoporosis and other osteolytic bone diseases.

## Data Availability Statement

The datasets generated for this study are available on request to the corresponding authors.

## Ethics Statement

The animal study was reviewed and approved by Guangzhou University of Chinese Medicine.

## Author Contributions

GH and LZ carried out the study design. GH, LZ, and XH conducted the experiments. XH and PS helped to analyze the data. ZC and WH provided experimental assistance. GH wrote the manuscript. JT and LC assisted data analysis. JX and XS supervised the overall project. JX and LC revised the manuscript.

## Funding

This study was supported by grants from Australian Health and Medical Research Council: APP1107828, APP1163933, APP1127156; National Natural Science Foundation of China: 81673999, 81704098; Guangdong Natural Science Funds for Distinguished Young Scholars: 2015A030306037; Science and Technology Planning Project of Guangdong Province: 2014A020221114. GH was a visiting scholar to UWA.

## Conflict of Interest

The authors declare that the research was conducted in the absence of any commercial or financial relationships that could be construed as a potential conflict of interest.

## Supplementary Material

Figure S1 Asiatic acid shows no effect on phosphorylation of P65 and P38.
