# Supplementary figures and images for: Asiatic Acid Inhibits OVX-Induced Osteoporosis and Osteoclastogenesis Via Regulating RANKL-Mediated NF-κb and Nfatc1 Signaling Pathways
Source: Front Pharmacol. 2020 Mar 27;11:331. doi: 10.3389/fphar.2020.00331 (PMC7120530; doi:10.3389/fphar.2020.00331)

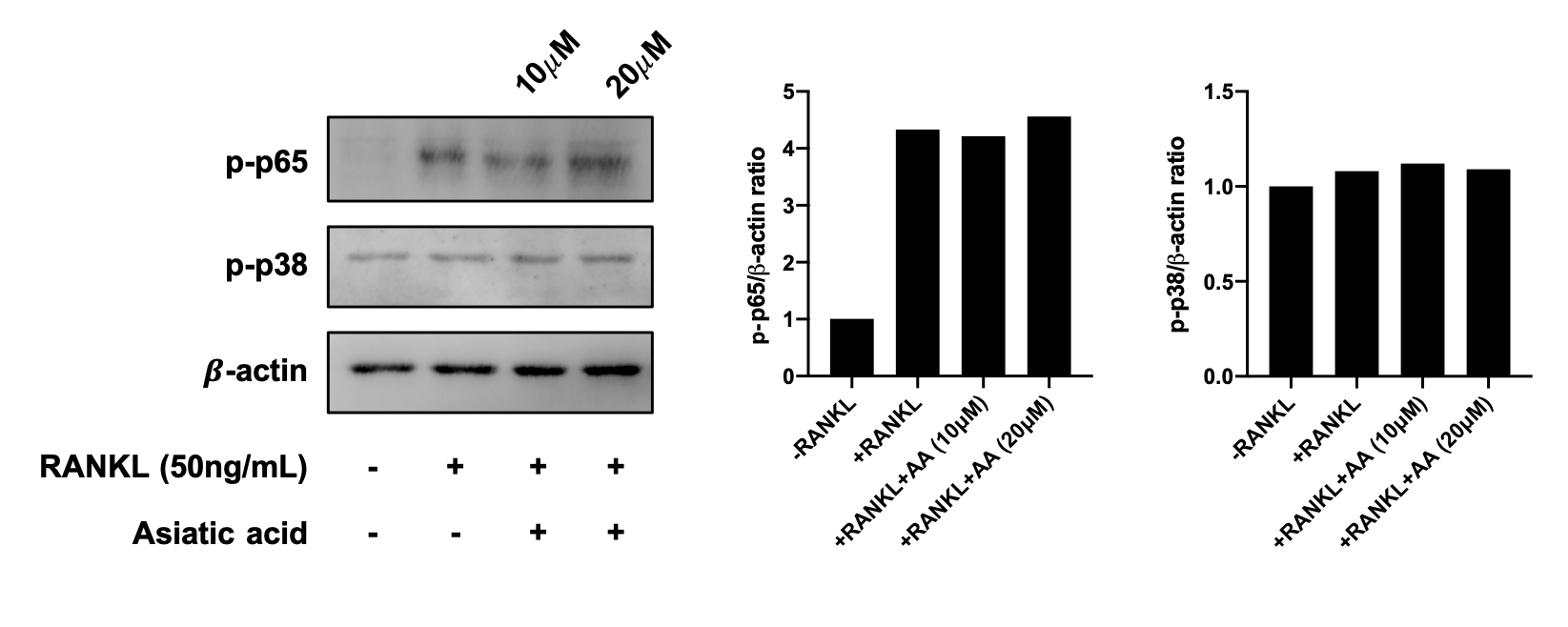

Supplement: Figure S1 — Asiatic acid shows no effect on phosphorylation of P65 and P38. [file Image_1.tif]
